# Evaluating Surveillance Indicators Supporting the Global Polio Eradication Initiative, 2011–2012

**Published:** 2013-04-12

**Authors:** Polio Eradication Dept, World Health Organization, Geneva, Switzerland, Div of Viral Diseases, National Center for Immunization and Respiratory Diseases, Global Immunization Div, Center for Global Health, Carolyn Sein

**Affiliations:** EIS Officer, CDC

The Global Polio Eradication Initiative (GPEI) was established in 1988 by the World Health Assembly to interrupt transmission of wild poliovirus (WPV); completion of this initiative was declared a programmatic emergency of public health in January 2012 (1,2). Polio cases are detected through surveillance for acute flaccid paralysis (AFP) with linked stool specimens tested for polioviruses (PVs) at accredited laboratories within the Global Polio Laboratory Network (GPLN). AFP surveillance findings are supplemented by testing sewage samples (environmental surveillance) collected at selected sites. Virologic data guide where targeted immunization activities should be conducted or improved. Key performance indicators are used to 1) monitor AFP surveillance quality at national and subnational levels to identify gaps where PV transmission could occur undetected; 2) provide evidence of where PV circulation has been interrupted; and 3) allow timely detection of an outbreak. Standardized surveillance indicators allow progress to be monitored over time and compared among countries (3). This report presents AFP surveillance performance indicators at national and subnational levels for countries affected by polio during 2011–2012, and trends in environmental surveillance, updating previous reports (4,5). In the 19 countries with transmission of PV (WPV and/or circulating vaccine-derived poliovirus [cVDPV]) during 2011–2012, national performance indicator targets were met in 12 (63%) countries in 2011 and 13 (68%) countries in 2012. Seven countries (37%) in 2011 had ≥80% of the population living in areas meeting performance indicators, increasing to nine countries (47%) in 2012. Performance indicators for timely reporting of PV isolation and characterization were met in four of six World Health Organization (WHO) regions in 2011 and five regions in 2012. To achieve global polio eradication, efforts are needed to improve and maintain AFP surveillance and laboratory performance.

## AFP Surveillance

AFP surveillance detects recent paralytic illness of any cause, including poliomyelitis caused by WPV or VDPV. The indicator used to determine if surveillance is sufficiently sensitive to detect PV circulation is the annual proportion of AFP cases that are negative for WPV and VDPV (nonpolio AFP [NPAFP]) among children aged <15 years. Countries in WHO regions certified as polio-free* should achieve an annual NPAFP rate of ≥1 case per 100,000 population aged <15 years; all other countries† should achieve annual rates of ≥2. To ensure sufficiently complete and reliable laboratory analysis, ≥80% of AFP cases should have two stool specimens collected ≥24 hours apart, within 14 days of paralysis onset, arriving in good condition at an accredited GPLN laboratory (adequate specimen). Because national data can mask heterogeneous subnational performance, the AFP surveillance indicators described in this report can be applied to subnational areas and assessed both individually and in combination. To assess population coverage in surveillance, the proportion of the national population residing in the subnational areas where both indicator targets are met is considered. Both national and subnational surveillance performance indicators are used to track GPEI progress.

In 2011, AFP surveillance detected WPV transmission in 16 countries: four countries with uninterrupted endemic transmission (Afghanistan, India, Nigeria, and Pakistan), three previously polio-free countries with reestablished transmission (Angola, Chad, and Democratic Republic of the Congo [DRC]), and nine countries with outbreaks following importation (Central African Republic [CAR], China, Côte d’Ivoire, Gabon, Guinea, Kenya, Mali, Niger, and Republic of the Congo) (Table 1) (6). In 2012, WPV transmission was detected in five countries (Afghanistan, Chad, Niger, Nigeria, and Pakistan); because the most recent confirmed WPV case in India had onset in January 2011, WHO removed India from the list of polio-endemic countries in February 2012.

In 2011, cVDPV cases were detected in seven countries (Afghanistan, DRC, Mozambique, Niger, Nigeria, Somalia, and Yemen) and in eight countries in 2012 (Afghanistan, Chad, DRC, Kenya, Nigeria, Pakistan, Somalia, and Yemen) (Table 1). All cVDPV outbreaks during 2011 and 2012 were type 2 except in Mozambique (type 1) and Yemen (type 3). cVDPV isolates detected in Kenya were genetically similar to cVDPV isolates detected in Somalia (7).

All 19 countries reporting PV transmission during 2011–2012 met the national target of an annual rate of ≥2 NPAFP cases per 100,000 population aged <15 years for both years (Table 1); the national target of ≥80% of AFP cases with adequate specimens was met by 12 (63%) countries in 2011 and 13 (68%) countries in 2012. The geographic distribution of subnational AFP surveillance quality indicators varied among countries with PV circulation (Table 1, Figure). In the African Region, ≥80% of the population lived in areas meeting both AFP surveillance quality indicators only in Nigeria in 2011, and in Angola, CAR, Kenya, and Nigeria in 2012. In addition, DRC, Mali, and Mozambique had substantial improvements in the proportion of the population living in areas meeting both AFP surveillance quality indicators from 2011 to 2012 (Table 1).

Six countries with PV circulation during 2011–2012 in the African Region had low proportions of subnational areas meeting the indicator for adequate specimen collection and low proportions of the population living in areas meeting subnational indicators in both years without substantial improvement (Chad, Republic of the Congo, Côte d’Ivoire, Gabon, Guinea, and Niger) (Table 1). Among neighboring countries with surveillance data available and without reported PV circulation during 2011–2012, quality gaps occurred in subnational AFP surveillance performance in 2012 (Figure).

## Environmental Surveillance

The sampling and testing of sewage can identify PV circulation in populations serviced by the sewage system and is used to complement AFP surveillance (8). Environmental surveillance has been established in two currently polio-endemic countries: Nigeria since 2011 (currently 11 sites in three states) and Pakistan since 2009 (currently 23 sites in four states), and in 22 countries without active WPV transmission: India (currently 15 sites in four states), Egypt (currently 34 sites in 11 cities), and multiple sites in 20 countries of the WHO European Region.

In Nigeria, WPV type 1 (WPV1) and VDPV type 2 (VDPV2) were isolated from sewage samples taken from four sites in Sokoto during March–December 2012, including periods when no PV was isolated from persons with AFP. In Kano, sewage sampling began in 2011 at three sites; VDPV2, WPV1, and WPV type 3 isolates were detected during January–September 2012.

In Pakistan, the number of environmental surveillance sampling sites increased from 17 in 2011 to 23 in 2012. WPVs have been isolated from sewage samples since testing began in 2009 from all major cities, even in the absence of confirmed WPV cases detected through AFP surveillance. Samples from Sindh consistently have yielded WPV isolates in the absence of associated WPV-positive AFP cases in the vicinity. In contrast, WPV cases were not detected in Quetta during the second half of 2012; environmental samples from the two Quetta sites also were negative during that period.

In Egypt, WPV1 was isolated from two samples collected in Cairo in December 2012; WPV1 was not detected from samples collected subsequently. The WPV1 sequences from these isolates were similar to WPV1 circulating in northern Sindh, Pakistan. WPV has not been detected in persons with AFP in Egypt since 2004.

## Global Polio Laboratory Network

GPLN consists of 146 WHO-accredited PV laboratories in all six WHO regions (5). GPLN member laboratories follow standardized protocols to 1) identify and isolate PV to confirm WPV cases; 2) differentiate the three PV serotypes (1–3), and WPV, Sabin-like PV,§ and VDPV (intratypic differentiation [ITD]); and 3) conduct genomic sequencing to monitor pathways of PV transmission by comparing the nucleotide sequence of the VP1 region of the genome from PV isolates (9,10). The two standard laboratory timeliness indicators for stool specimen processing are to report ≥80% ITD results within 7 days of receipt of specimen and ≥80% of sequencing results within 7 days of receipt of specimen. The independent programmatic indicator standard is to report ITD results for ≥80% of isolates within 60 days of paralysis onset of persons with AFP cases; this indicator takes into account the entire interval from onset of paralysis through case notification, investigation, and specimen collection, transport, and testing (the WHO Eastern Mediterranean Region uses a 45-day timeframe). In addition to timeliness, the accuracy and quality of laboratory testing are monitored through an annual accreditation program of onsite reviews and proficiency testing (9). During 2011–2012, GPLN laboratories in five WHO regions met timeliness indicators for PV isolation. Reporting indicators for onset to ITD results were met in four WHO regions in 2011 and in all WHO regions in 2012 (Table 2). GPLN tested 215,629 stool specimens in 2012, compared with 206,981 specimens in 2011 (4% increase). In 2012, a total of 395 WPV isolates were detected from all sources (AFP and environmental sample specimens), compared with 1,570 WPV isolates in 2011 (a 75% decrease). In addition, 7,349 Sabin-related PV and 125 VDPV isolates were detected in 2012, compared with 8,569 Sabin-related PV and 93 VDPV isolates detected in 2011.

During 2012, genomic sequencing identified two WPV1 genotypes and one WPV3 genotype in the African Region: West Africa-B1 (WEAF-B1) type 1 genotype was detected in Nigeria, Niger, and Chad; WEAF-B2 type 1 genotype and WEAF-B type 3 genotype only were detected in Nigeria. In the WHO Eastern Mediterranean Region, South Asia (SOAS) type 1 and SOAS type 3 genotypes were detected in 2012. When genomic sequencing detects >1.5% nucleotide sequence divergence from previously identified PV isolates, this highlights quality gaps in AFP surveillance. Sequence analysis indicated that chains of WPV transmission had been missed by AFP surveillance during 2012 in Afghanistan, Chad, Nigeria, Pakistan, and Niger; chains of VDPV transmission also were missed in Nigeria and Somalia.

### Editorial Note

Notable gains in interrupting WPV transmission have occurred since GPEI was declared a programmatic emergency in January 2012; the number of countries with WPV transmission decreased from 16 in 2011 to five in 2012, and reported WPV cases decreased from 650 to 223, with WPV transmission now primarily in localized “sanctuaries.”¶ AFP surveillance performance indicators met certification-level quality in the majority of countries with PV circulation during 2011–2012 including polio-endemic countries, and improved during this period in Angola, CAR, and DRC; however, critical surveillance gaps remain in parts of Cameroon, Chad, and Niger that border areas of Nigeria with ongoing WPV transmission, and at subnational levels in multiple countries.

What is already known on this topic?

Progress of the Global Polio Eradication Initiative (GPEI) is monitored through surveillance of acute flaccid paralysis (AFP) cases, laboratory surveillance to test stool specimens for polio viruses (PVs), typing and sequencing tests to track PV transmission, and environmental surveillance of sewage samples of PV at selected sites. Standardized indicators enable monitoring of progress over time and comparison between countries.

What is added by this report?

Progress has been made since polio eradication was declared a global public health programmatic emergency in 2012. During 2011–2012, wild poliovirus (WPV) case numbers and the number of countries with WPV transmission decreased. However, in 2012, only 63% of countries with WPV circulation met national AFP surveillance indicator targets, compared with 62% in 2011. During 2011–2012, the number of countries with PV transmission with ≥80% of their populations living in areas meeting surveillance indicators increased from seven of 19 (37%) to nine of 19 (47%).

What are the implications for public health importance?

PV transmission can be undetected in areas where gaps in AFP surveillance quality exist, and this trend has been confirmed through laboratory analysis and environmental surveillance. Improving sensitivity for PV detection will involve expanding environmental surveillance activities and strengthening AFP performance, particularly at the subnational level, with ongoing supervision and monitoring of active surveillance at the health facility level.

Environmental sampling continues to complement AFP surveillance in determining areas where PV circulates. GPEI plans to expand environmental surveillance in areas at highest risk for WPV transmission or cVDPV emergence, with consideration of site feasibility and laboratory capacity (2). Strengthening AFP surveillance becomes increasingly important to detect low-level WPV circulation in its last remaining foci of transmission to target intensified activities, promptly detect any new outbreaks, and eventually achieve, document, certify, and maintain regional polio-free status.

Regional certification of polio-free status only occurs when all member states demonstrate the absence of WPV transmission for 3 consecutive years with surveillance meeting performance targets. Global certification occurs only when all regions are certified polio-free, maintain certification-standard surveillance, and implement posteradication containment measures (2). The detection of PV in some countries (e.g., Afghanistan, Nigeria, Pakistan, and Somalia) that is highly diverged from previously identified PV isolates indicates that WPV or VDPV transmission remained undetected by AFP surveillance even when AFP performance indicators were met at the state/provincial level (6). For these reasons, increased emphasis will be placed on activities to ensure that AFP surveillance performance is maintained and improved at all administrative levels throughout each country in 2013. This can be accomplished by 1) tracking implementation of recommendations after surveillance reviews, 2) continuous monitoring of indicators at all administrative levels, 3) retraining staff, 4) improving timely collection and appropriate transportation of stool specimens, and 5) enhancing supervision. Ongoing supervision of active surveillance at health facilities also is needed to ensure optimal surveillance performance, with special attention to populations with a high risk for undetected PV transmission (e.g., mobile and displaced populations). In countries with large populations (e.g., Nigeria and Pakistan), surveillance performance needs to be closely monitored at lower administrative levels (e.g., districts).

The GPEI Polio Eradication and Endgame Strategic Plan for 2013–2018** includes specific efforts to 1) interrupt all PV transmission, 2) certify polio eradication, 3) withdraw OPV, and 4) strengthen routine immunization and surveillance systems as part of the legacy of GPEI. The strategic plan will be submitted to the World Health Assembly in May 2013 to reinvigorate the commitment of countries and other GPEI partners toward polio eradication.

## Figures and Tables

**FIGURE:**
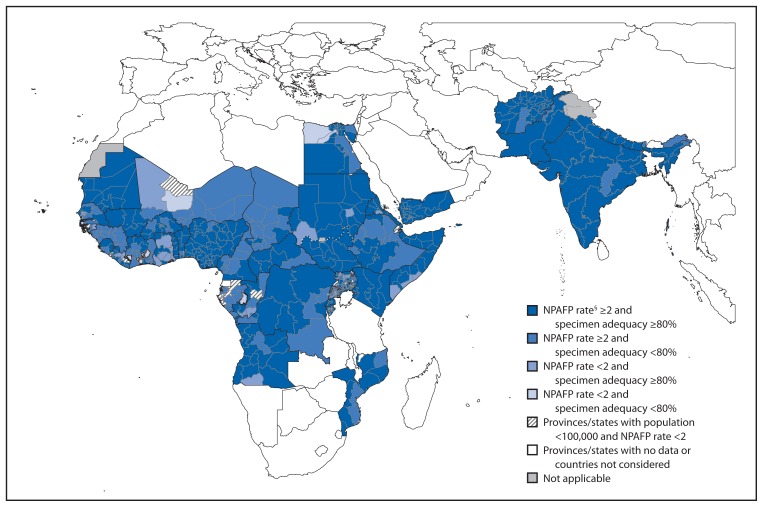
Combined performance indicators for the quality of acute flaccid paralysis (AFP) surveillance* in subnational areas (states and provinces) of 16 polio-affected countries and neighboring countries, 2012^†^ **Abbreviation:** NPAFP = nonpolio AFP. * The Global Polio Eradication Initiative 2010–2012 strategic plan sets the following targets for countries with current or recent wild poliovirus transmission and their states/provinces: 1) NPAFP detection rate of two or more cases per 100,000 persons aged <15 years, and 2) adequate stool specimen collection from ≥80% of AFP cases, with specimen adequacy defined as two specimens collected ≥24 hours apart, both within 14 days of paralysis onset, shipped on ice or frozen packs, and arriving in good condition (without leakage or desiccation) at a World Health Organization–accredited laboratory. ^†^ Data are for AFP cases with onset during 2012, reported as of February 13, 2012. ^§^ Per 100,000 persons aged >15 years.

**TABLE 1:** National and subnational acute flaccid paralysis (AFP) surveillance indicators and number of confirmed wild poliovirus (WPV) and circulating vaccine-derived poliovirus (cVDPV) cases, by World Health Organization (WHO) region and polio-affected country, 2011 and 2012*

	2011							2012						
		
WHO region†/Country	No. of AFP cases	National NPAFP rate§	% subnational areas with NPAFP rate ≥2¶	National % AFP cases with adequate specimens**	% subnational areas with ≥80% adequate specimens	% population in areas meeting both indicators††	No. of confirmed WPV cases (No. of cVDPV cases)§§	No. of AFP cases	National NPAFP rate§	% subnational areas with NPAFP rate ≥2¶	National % AFP cases with adequate specimens**	% subnational areas with ≥80% adequate specimens	% population in areas meeting both indicators	No. of confirmed WPV cases (No. of cVDPV cases)§§
**African**	16,636	4.4	—	88	—	—	350 (48)	18,032	4.8	—	90	—	—	127 (40)
Angola†††	256	2.3	56	91	89	43	5	319	3.1	94	90	94	95	—
CAR***	142	6.0	100	80	71	68	4	124	6.3	100	85	86	88	—
Chad†††	465	5.7	100	75	39	33	132	418	6.7	100	71	22	20	5 (12)
Côte d’Ivoire***	511	5.1	95	64	0	0	36	331	3.5	74	77	29	25	—
DRC†††	2,222	4.9	100	79	27	33	93 (11)	1,858	4.4	100	83	64	70	(17)
Gabon***	30	2.9	0	60	33	10	1	25	2.4	0	12	0	0	
Guinea***	205	3.8	100	68	0	0	3	187	3.3	100	62	0	0	—
Kenya***	559	3.0	88	84	75	49	1	715	4.2	100	91	100	100	(3)
Mali***	210	2.7	100	84	67	64	7	266	3.4	75	91	75	77	—
Mozambique	314	2.7	80	87	80	59	(2)	337	3.1	100	88	70	77	—
Niger***	319	4.0	88	73	25	20	5 (1)	365	4.5	100	68	0	0	1
Nigeria†††	6,099	7.9	100	93	100	100	62 (34)	7,223	8.7	100	94	97	96	122 (8)
Republic of the Congo***	93	3.1	60	75	55	20	1	62	2.9	50	76	55	16	—
**Eastern**	11,742	5.7	—	90	—	—	278 (19)	10,956	5.2	—	91	—	—	95 (27)
**Mediterranean**														
Afghanistan†††	1,831	10.0	100	92	91	91	80 (1)	1,829	10.2	100	92	94	91	37 (8)
Pakistan†††	5,767	7.1	100	88	88	95	198	4,878	6.3	100	89	88	98	58 (16)
Somalia	172	3.2	94	98	95	81	(9)	148	2.8	75	98	100	56	(1)
Yemen	386	3.4	100	91	95	93	(9)	477	4.0	100	93	95	98	(2)
**South-East Asia**	65,331	12.1	—	85	—	—	1	66,067	12.2	—	87	—	—	0
India†††	60,540	13.5	91	84	82	89	1	60,994	14.0	100	87	86	97	—
**Western Pacific**	7,303	2.1	—	90	—	—	21	7,569	2.2	—	91	—	—	0
China***	6,182	2.8	81	94	97	91	21	6,181	2.8	77	94	97	87	—
**Total**	**101,012**	**5.6**	**—**	**88**	**—**	**—**	**650 (67)**	**102,624**			**90**			**223 (67)**

**Abbreviations:** NPAFP = nonpolio AFP; CAR = Central African Republic; DRC = Democratic Republic of Congo.

* Data as of February 5, 2013.

† Regional NPAFP rates use United Nations Development Program population estimates as denominators; these tend to be higher than country rates, which use their summed subnational population estimates as denominators. Regional data available at http://apps.who.int/immunization_monitoring/en/diseases/poliomyelitis/case_count.cfm.

§ Per 100,000 persons aged <15 years.

¶ For subnational areas (states and provinces) with populations >100,000.

** Standard WHO target is adequate stool specimen collection from ≥80% of AFP cases, in which two specimens are collected ≥24 hours apart, and within 14 days of paralysis onset, and arriving in good condition (received by reverse cold chain and without leakage or desiccation) in a WHO-accredited laboratory. Stool adequacy proportions from the WHO regions and China do not include criteria of good stool specimen condition.

†† For all subnational areas regardless of population size.

§§ cVDPV is associated with two or more cases of AFP. Kenya cVDPVs in 2012 are linked to the Somalia outbreak. VDPV type 2 cases with greater than or equal to six nucleotide differences from AFP sources. Nigeria data include one case in 2011 with WPV1/cVDPV mixture.

¶¶ Countries with reestablished WPV transmission.

*** Countries with WPV outbreaks.

††† Countries with endemic WPV transmission.

**TABLE 2:** Number of poliovirus (PV) isolates from stool specimens of persons with acute flaccid paralysis and timing of results, by World Health Organization (WHO) region, 2011 and 2012*

WHO region	2011						2012					
	
	No. of specimens	No. of PV isolates			% PV isolation results on time¶	% ITD results within 60 days**	No. of specimens	No. of PV isolates			% PV isolation results on time¶	% ITD results within 60 days**
	
		Wild	Sabin†	cVDPV§				Wild	Sabin†	cVDPV§		
African	36,942	1,035	2,476	46	91	86	39,710	221	2,629	43	95	93
Americas	1,762	0	36	1	61	**—**	1,926	0	31	0	77	100
Eastern Mediterranean	23,011	512	807	17	98	97	26,626	174	930	71	94	99
European	3,270	0	77	7	96	78	3,167	0	66	2	96	88
South-East Asia	127,543	2	4,907	15	97	98	129,106	0	3,470	1	98	100
Western Pacific	14,453	21	266	7	97	86	15,094	0	223	8	98	84
**Total**	**206,981**	**1,570**	**8,569**	**93**	**90**	**74**	**215,629**	**395**	**7,349**	**125**	**93**	**94**

**Abbreviations:** cVDPV = circulating vaccine-derived poliovirus; ITD = intratypic differentiation.

* Data as of February 13, 2013 (Uzbekistan excluded, no data provided).

† Either concordant Sabin-like results in ITD test and VDPV screening, or <1% sequence difference compared with Sabin vaccine virus (<0.6% for PV type 2).

§ For PV types 1 and 3, 10 or more VP1 nucleotide differences from the respective PV; for PV type 2, six or more VP1 nucleotide differences from Sabin type 2 PV.

¶ Results reported within 14 days for laboratories in the following WHO regions: African, Americas, Eastern Mediterranean, and South-East Asia, and Western Pacific (not including China). Results reported within 28 days for the European Region and China.

** Results reported within 60 days of paralysis onset for all WHO regions except Eastern Mediterranean Region, which reported within 45 days of paralysis onset.

## Footnotes

* American, European, and Western Pacific regions.

† Countries in the African, Eastern Mediterranean, and South-East Asian regions.

§ Demonstrating <6 nucleotide changes from Sabin OPV strains for PV type 2, and <10 changes from Sabin OPV strains for PV types 1 and 3.

¶ Discrete geographic locations with large numbers of missed children where the virus has ample opportunity to circulate.

** Current draft available at http://www.polioeradication.org/resourcelibrary/strategyandwork.aspx.
